# Host iron status and erythropoietic response to iron supplementation determines susceptibility to the RBC stage of falciparum malaria during pregnancy

**DOI:** 10.1038/s41598-017-16896-z

**Published:** 2017-12-15

**Authors:** Morgan M. Goheen, Amat Bah, Rita Wegmüller, Hans Verhoef, Bakary Darboe, Ebrima Danso, Andrew M. Prentice, Carla Cerami

**Affiliations:** 1 Department of Microbiology and Immunology, University of North Carolina School of Medicine, Chapel Hill, NC USA; 2 Nutrition Theme, MRC Unit The Gambia, MRC International Nutrition Group, Keneba, The Gambia; 3 London School of Hygiene & Tropical Medicine, London, UK; 4 Division of Human Nutrition and Cell Biology and Immunology Group, Wageningen University, Wageningen, The Netherlands

## Abstract

Anaemia and malaria are both common in pregnant women in Sub-Saharan Africa. Previous evidence has shown that iron supplementation may increase malaria risk. In this observational cohort study, we evaluated *P. falciparum* pathogenesis *in vitro* in RBCs from pregnant women during their 2nd and 3rd trimesters. RBCs were collected and assayed before (n = 327), 14 days (n = 82), 49 days (n = 112) and 84 days (n = 115) after iron supplementation (60 mg iron as ferrous fumarate daily). *P. falciparum* erythrocytic stage growth *in vitro* is reduced in anaemic pregnant women at baseline, but increased during supplementation. The elevated growth rates parallel increases in circulating CD71-positive reticulocytes and other markers of young RBCs. We conclude that *Plasmodium* growth *in vitro* is associated with elevated erythropoiesis, an obligate step towards erythroid recovery in response to supplementation. Our findings support current World Health Organization recommendations that iron supplementation be given in combination with malaria prevention and treatment services in malaria endemic areas.

## Introduction

Malaria and anaemia are two common health problems facing pregnant women in Sub-Saharan Africa. Anaemia is found in 43% of pregnant women worldwide^1^. Iron deficiency accounts for 50–75% of anaemia cases, and is thought to be largely due to inadequate diet and increased nutritional requirements during pregnancy^2^. Pregnant women are also at increased risk of *P. falciparum* malaria infection. Susceptibility peaks during the second trimester and risk remains elevated for up to 60 days post-partum. It is highest in primagravida women, however women of all gravidities have increased susceptibility to malaria. There are several possible explanations: (1) pregnant women are more attractive to mosquitoes^3^; (2) pregnant women have higher rates of erythropoiesis and higher percentages of young red blood cells (RBCs) which are preferred by *P. falciparum* ^4,5^; and (3) parasitized RBCs accumulate in the placenta thus reducing immune surveillance associated with normal splenic circulation^6^. Specifically, it is well known that parasites infecting pregnant women express a particular protein encoded by *var2csa* that binds chondroitin sulphate A expressed on placental syncytiotrophoblasts which allows for placental sequestration of infected RBCs^7^. Primagravida women who have not been immunologically exposed to *var2csa* protein products are particularly vulnerable^8^. No matter what the mechanism of increased susceptibility is, maternal infection has severe consequences for both the mother and fetus^9^.

World Health Organization (WHO) guidelines recommend 30 to 60 mg elemental iron and 400 mg folic acid daily throughout pregnancy. These recommendations are based on evidence showing that daily iron and folic acid reduces the risk of low birth weight, preterm birth, maternal anaemia and maternal iron deficiency^10^. However, these recommendations have been questioned recently by several findings: (1) iron deficiency and anaemia are associated with protection against malaria in pregnant women and children^11–16^; and (2) iron supplementation in children has been associated with increased mortality and of risk of infectious diseases, including malaria^17–22^.

Multiple studies have investigated the safety of iron supplementation of children in malaria endemic areas. However, there has been less focus on the safety of iron supplementation of pregnant women, mainly because WHO guidelines for pregnant women suggest the use of intermittent preventive treatment for malaria with sulfadoxine-pyrimethamine (IPTp-SP) in areas with moderate to high malaria transmission. Starting as early as possible in the second trimester, IPTp-SP is recommended at each scheduled antenatal care visit^23^. However, implementation of IPTp is low; in fact only about 25% of pregnant women in Sub-Saharan Africa received two or more doses of IPTp^24^. Hence, the issue of safety of iron supplements for pregnant women living in malaria endemic areas remains important.

In this study, we have used *in vitro* malaria growth assays in RBCs from pregnant women as a proxy for malaria susceptibility. Use of *in vitro* assays allowed us to assess the impact of host iron supplementation in the absence of the confounding effects from IPTp-SP or the host immune system. We have systematically assessed *P. falciparum* growth *in vitro* in RBCs drawn from pregnant women before, during, and after 12 weeks of iron supplementation, which was initiated during the 2^nd^ trimester of pregnancy. The women in this study did receive IPTp-SP, however they donated blood for the malaria *in vitro* growth assays at each of the scheduled visits prior to ingestion of the monthly dose. Similar to our research involving *in vitro P. falciparum* growth assays in RBCs longitudinally collected from anaemic children undergoing iron supplementation^25^, here we observe that parasite growth correlates with host haemoglobin status at baseline. We also observe parasite growth increases with iron supplementation, paralleling increases in circulating young RBCs, and potentially representing a period of increased malaria susceptibility following iron supplementation of pregnant women.

## Materials and Methods

### Subject recruitment

The pregnant women for this observational cohort study were recruited from the reference (Gambian government standard of care) arm of a randomized trial testing the efficacy and safety of a hepcidin-guided screen-and-treat strategy for combatting anaemia (see published protocol for full details including sample size calculations and randomization^26^) and were 18–45 years old with estimated 14–22 weeks gestation at recruitment. Recruitment occurred from June 2014–March 2016 in the Kiang West and Jarra East regions of rural Gambia^26^. (Note we also included pregnant women in the other two arms of this trial, but only for observation at baseline, pre-randomization/pre-intervention.) As per current WHO and Gambian government recommendations, pregnant women in the reference arm received 60 mg/d iron as ferrous fumarate and 400 µg folic acid, given within a micronutrient capsule (UNIMMAP). Field workers visited the women frequently (twice weekly) in order to check their health status and deliver the supplements. For baseline population characteristics, see Supplemental Table 1.

### UNIMMAP capsules

The iron supplement used in this trial was included in a micronutrient mixture (UNICEF/WHO/UNU international multiple micronutrient preparation (UNIMMAP)) produced by the DSM Company (South Africa). It contains: 60 mg iron, 400 µg folic acid, vitamin A (800 µg RE), vitamin D (200 IU), vitamin E (10 mg), thiamine B1 (1.4 mg), riboflavin B2 (1.4 mg), niacin B3 (18 g), vitamin B6 (1.9 mg), vitamin B12 (2.6 µg), vitamin C (70 mg), zinc (15 mg), iodine (150 µg), selenium (65 µg), and copper (2 mg).

### Blood samples for parasite assays

**Figure 1:**
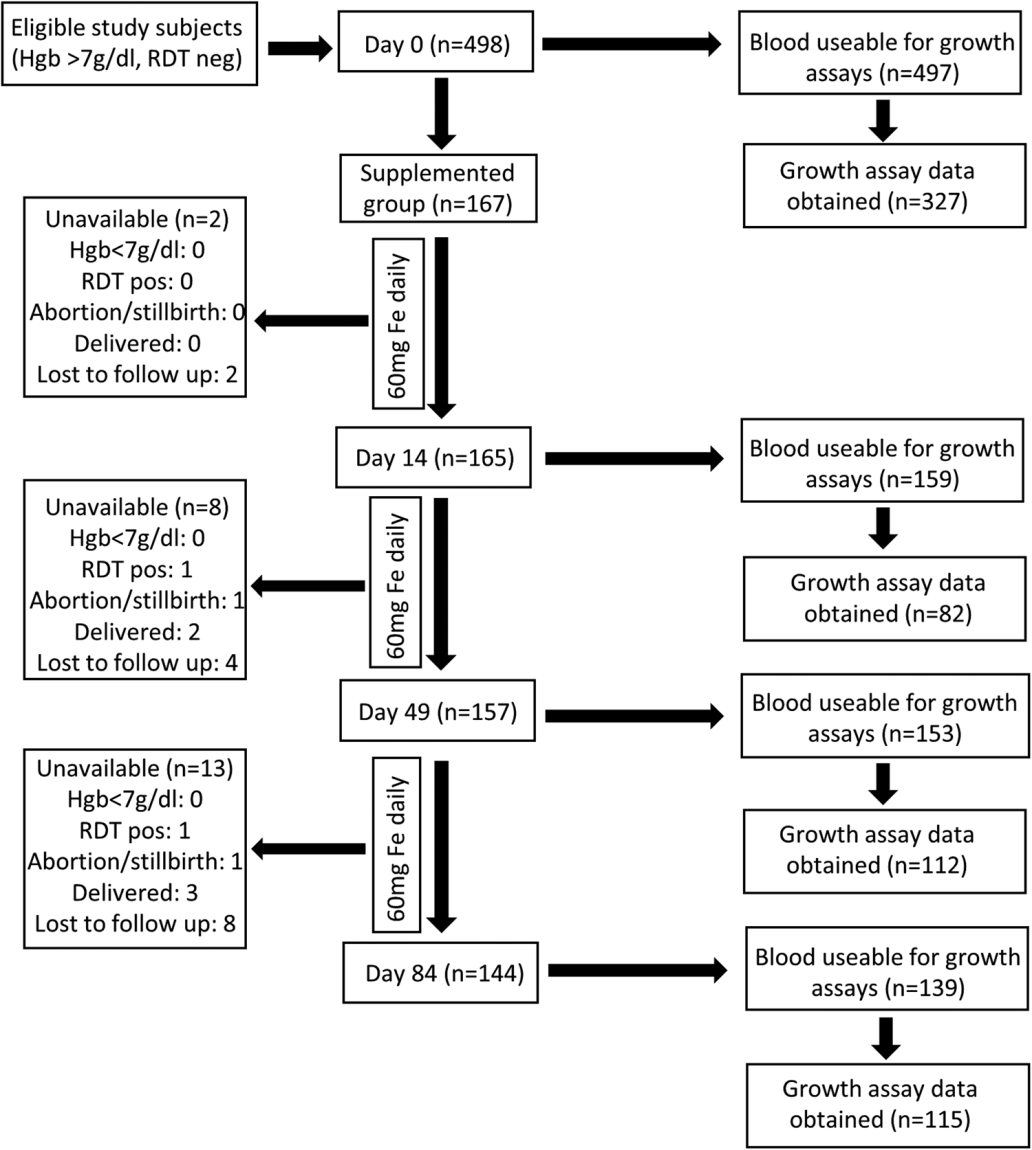
Description of subjects and flow chart of sample collection and assays performed. Blood samples for haematological, biochemical, and parasite growth analyses were drawn at Day 0, as well as Days 14, 49, and 84 for those taking daily iron. A full haematology panel was measured in EDTA-stabilized blood (Medonic M20M GP). We also assayed ferritin, soluble transferrin receptor (sTfR), serum iron, transferrin saturation (TSAT), C-reactive protein (CRP), alpha 1-acid glycoprotein (AGP) (Cobas Integra 400 plus); and hepcidin (Hepcidin-25 (human) EIA Kit (Bachem)). Genotyping for haemoglobinopathies was performed using haemoglobin electrophoresis. For malaria assays, 2.5 mL of venous blood was drawn directly into microvette tubes containing CPDA-1 (Sarstedt, Germany). Unavailable donors include safety exclusion (Hgb < 7 g/dl or positive malaria test (RDT positive)), those who delivered prematurely or aborted, or general loss to follow up (withdrawal and travel). Failure to collect blood from subjects (e.g. from phlebotomy failure, subject moved or withdrew, delivered, or became significantly ill) was 0.2% at Day 0, 4.8% at Day 14, 8.4% at Day 49, and 16.8% at Day 84. RBCs from study subjects were evaluated with *in vitro P. falciparum* growth assays (using strain FCR3-FMG) as a proxy measure for malaria susceptibility. In order to standardize the growth assays, control for inter-assay variability and variability between parasite preparations, assays on clinical samples were run in parallel with and reported relative to growth assays done using RBCs from non-anaemic donors. Each available blood sample at every time point was subjected to growth assays but not all produced growth data, as some blood was unusable (e.g. clotted, hemolysed, or contaminated). Further growth data exclusions (e.g. parasites died or control blood did not provide a readable output for comparison) do not represent population sampling bias, as subject characteristics are the same between those with and without corresponding growth data (Supplemental Table 2).

Venous blood was collected at Days 0 (baseline), 14, 49 and 84 during 12wks of iron supplementation (Fig. 1). We compared subject characteristics of those whose blood was and was not able to be used for malaria growth rate data to ensure no sampling bias occurred (Supplemental Table 2).

### *P. falciparum* culture

Parasite lines FCR3-FMG (MR4, MRA-736) and 3D7 (MR4, MRA-102) were routinely cultured in RBCs from healthy donors using standard methods^4^. 3D7 knockout parasite strains ΔPfRh1^27^, ΔPfRh2a, and ΔPfRh2b^28^ were kindly provided by Tony Triglia (WEHI, Australia).

### Growth assay

*In vitro* growth was assessed in fresh, washed RBCs as in^4^ for 96 h (performed in triplicate for RBCs from each study participant). RBCs from healthy, non-pregnant, adult iron replete donors of normal haemoglobin genotype and G6PD status not undergoing iron supplementation served as controls. Growth rates represent final 96 h parasitaemia divided by initial 0 h parasitaemia, analysed by flow cytometry^4^.

### Quantification of CD71-positive reticulocytes

CD71-positive reticulocytes in fresh RBCs were counted using PE-conjugated anti-human CD71 antibody (Clone M-A712, BD) and isotype control (Clone G155–178, BD), and analysed by flow cytometry for CD71-positive reticulocyte percentage relative to non-anaemic control as in^29^.

### RBC barcoding invasion assay

The assay was performed and analyzed as in^25^ using two different concentrations of CellTrace Far Red DDAO (Invitrogen Life Technologies/Molecular Probes): 1 µM (high) or 0.1 µM (low). Differentially labeled RBCs were combined in equal density in triplicate into a 96 well plate and seeded with MACS purified (Miltenyi Biotec) late stage parasites to achieve 1.5–2% pRBCs in 1% hematocrit. Plates were maintained for 12–18 hours to allow for schizont rupture and subsequent invasion of labeled RBCs. Invasion into high and low DDAO RBCs was detected by DNA dye Sybr Green I, and analyzed by flow cytometry as in^1^, and the ratio of the prevalences of parasitized high to low DDAO RBCs was calculated to generate the “Susceptibility Index”. The parasite strains used were parent strain 3D7 and knockout strains ΔPfRh1, ΔPfRh2a, and ΔPfRh2b, all assayed in parallel for invasion into the same donor RBCs from non-anemic controls and anemic, pregnant subjects (n = 15). This allowed for comparison of invasion deficits of parent and transgenic parasite strains into anemic RBCs versus non-anemic RBCs.

### Attachment assay

Assays and analysis of merozoite attachment to anemic versus non-anemic RBCs were performed based on^30^. In detail, tightly synchronized parasite cultures (15 ml of 4% hematocrit cultures containing 5% parasitized RBCs) were treated with 50 U/ml of heparin as trophozoites. Approximately 12 h later, upon visualization of schizonts by Geimsa stain, schizonts were purified by MACS. Resulting schizont concentration was quantified using Sybr Green stain and flow cytometry, and schizonts were incubated in ACM containing 50 U/ml heparin under standard culture conditions to allow for 3–4 h of recovery. RBC concentrations of the donor samples were also quantified by flow cytometry. To set up the assay, schizonts were washed twice with 5 ml heparin-free ACM and distributed equally amongst triplicate wells (of a 96 well plate) for each assay condition. RBCs from anemic or non-anemic donors were then added to appropriate wells to bring the final concentration of schizonts to 10%. ACM with either no drug (to assay merozoite invasion), 1 µM Cytochalasin D (to assay merozoite attachment), or 200 U/ml heparin (to block both invasion and attachment), was added to appropriate wells and plates were incubated under standard culture conditions. At each time point (0, 3, and 7 h), 50 µl of each drug treatment condition from each blood sample was aliquoted in triplicate into 200 µl of fixative (PBS containing 0.116 M sucrose and 2% glutaraldehyde), with overnight fixation at 4°C before staining with DNA dye Sybr Green I and analysis by flow cytometry. Giemsa stains were also made from each time point and condition in order to confirm that attachment and invasion events were prevented by the various treatments and to verify that new events (in the ring region of the flow cytometry plot for DNA quantification) could correctly be counted as either invasion or attachment events. Flow cytometry analysis allows the use of DNA levels in parasite cultures to distinguish seeded schizonts present apart from new invasion or attachment events which occur at 3 and 7 h. To calculate attachment or invasion rates for each blood sample, the percent of new events was compared to the starting concentration of schizonts under each condition, then expressed relative to new events occurring in RBCs from healthy iron replete controls.

### RBC surface marker assessment

RBC surface protein levels were determined by staining with fluorescently tagged antibodies and analyzed by flow cytometry. The following antibodies were used: for CD35 (Mouse Anti-Human CD35 Clone E11 (BD) primary 1:2000; Alexa Fluor® 647 Rabbit Anti-Mouse IgG secondary 1:2000); for CD47 (Mouse Anti-Human CD47 Clone B6H12 primary 1:2000; Alexa Fluor® 647 Rabbit Anti-Mouse IgG secondary 1:500); for CD55 (Mouse Anti-Human CD55-PE conjugate Antibody NaM16-4D3 (Santa Cruz Biotechnologies) 1:10); for CD147 (Mouse Anti-Human CD147 Clone HIM6 (BD) primary 1:500; Alexa Fluor® 647 Rabbit Anti-Mouse IgG secondary 1:500); for Glycophorin A (GPA) (primary Rabbit Anti-Human CD235a/Glycophorin A (Thermo Fisher Scientific/Pierce) 1:500 and secondary Alexa Fluor® 647 Goat Anti-Rabbit 1:2000); for sialic acid residues (Wheat Germ Agglutinin Alexa Fluor® 488 Conjugate (Thermo Fisher Scientific/Molecular Probes) 1:2000); and for C3b deposition (primary Mouse Anti-Human Complement C3b Antibody 10C7 (Thermo Fisher Scientific) 1:200; Alexa Fluor® 647 Rabbit Anti-Mouse IgG secondary 1:500). The Relative Fluorescence Intensity (RFI) of each surface marker for each study sample was calculated using the MFI values (corrected for background), relative to MFI in the non-anemic donor.

### Flow cytometry analysis

Flow cytometry was performed onsite at MRCG in Keneba using a BD Accuri C6 flow cytometer. Channels and probes used included: SYBR Green I, FITC, and Alexa Fluor® 488 (488 nm excitation with a 530/30 nm band pass emission filter, detector FL1); PE (488 nm excitation with a 585/40 nm band pass emission filter, detector FL2); and CellTrace Far Red DDAO and Alexa Fluor® 647 (640 nm excitation with a 675/25 nm band pass emission filter, detector FL4). Detector gain setting changes and compensation were not necessary with this configuration. Accuri C6 data was collected and analyzed with Accuri software (BD Accuri CSampler Analysis Software). Linear amplification of forward scatter was used to set event threshold in order to exclude cell debris, microparticles and doublets. For all experiments, samples were diluted to 0.001–0.002% hematocrit and ≥100,000 total events were collected.

### Statistics

All experiments were done in triplicate. Growth rates, invasion assays, and haematological data were compared by two-tailed Student’s *t*-test, one-way ANOVA, and/or 95% confidence interval (CI) values using GraphPad Prism 5.

### Multivariate modelling

We employed linear regression to estimate the effect of host haematological factors on *in vitro* parasite growth rates. First, bivariate associations and their respective 95% CI were calculated between growth rates and haematological and patient characteristics at Day 0. We then used multivariate linear regression – specifically, we estimated the independent effects of haemoglobin and haemoglobin genotype on growth rate along with their respective 95% CI. We used directed acyclic graphs to identify potential confounders and controlled for them in our modelling approach^31^. An *a priori* alpha of 0.05 was used to determine statistical significance. Modelling was performed using R software (RStudio Version 0.99.902).

### Ethics approval

The trial from which pregnant women were recruited and the procedures described here were approved by the MRC Unit The Gambia (MRCG) Scientific Coordinating Committee and MRCG/Gambian Government Joint Ethics Committee (SCC 1357) and the UNC IRB (13–3068). We confirm that all experiments were performed in accordance with relevant guidelines and regulations for human subjects research. Participants were given a full description of the study and provided written signed, informed consent. The Clinical Trials Network registration number is ISRCTN21955180, and the trial was registered on Apr 4, 2014.

### Role of the funding source

None of the funding sources had a role in study design, data collection or interpretation, writing of the manuscript, or the decision to submit for publication. The corresponding author had full access to all the data included in the study and assumed final responsibility for the decision to publish; all authors reviewed the report and agreed to submit for publication.

## Results

### Description of study population at baseline

The study population consisted of 498 healthy pregnant women identified in the Jarra West and Kiang East regions of rural Gambia who were estimated to be at 14–22 weeks gestation during recruitment, fit all enrolment criteria, and consented to participate^26^. It is difficult to assess anaemia and iron deficiency in pregnant women^32^. It is complicated by changes in haematological dynamics during pregnancy and, in our setting, the backdrop of high levels of environmental inflammatory stimuli. The WHO defines anaemia in pregnant women as haemoglobin <11 g/dL^33^. During the second trimester of pregnancy, the maternal plasma volume expands much more rapidly than the RBC mass, resulting in hemodilution and a drop in haemoglobin concentrations by 0.5 g/dl in normal pregnancies (reviewed in^2^). The US Center for Disease Control defines anaemia as haemoglobin <10.5 g/dl in the 2nd trimester^34^, and the US Institute of Medicine recommends lowering haemoglobin reference values by 0.8 g/dl for African American women^35^. We therefore assessed anaemia in our study population by multiple haemoglobin standards. We found 54.4% of our participants had Hgb < 11 g/dl; 39.4% had Hgb < 10.5 g/dl; 17.5% had Hgb < 9.7 g/dl.

To further assess iron status, we classified our participants using two different definitions of iron deficiency: 1) ferritin < 12 ng/ml or ferritin 12–30 ng/ml and CRP > 5 mg/l; and 2) hepcidin < 2.5 ng/ml^36^. At baseline, 24.8% of the participants had ferritin < 12 ng/ml and 8.3% had ferritin 12–30 ng/ml and CRP > 5 mg/l. Thus, 33.1% of the participants fit the definition of iron deficiency based on ferritin measurements. Finally, using hepcidin to define iron deficiency, at baseline 54.2% of the participants were iron deficient (Table 1). For further characteristics of study subjects at enrolment, see Table 1 and Supplemental Tables 1 and 2.

**Table 1:** Blood, inflammatory, and iron parameters of donors whose RBCs were used for parasite growth assays before (Day 0), during (Days 14 and 49), and after (Day 84) iron supplementation.

Variable	Normal 2^nd^ Trimester	Normal 3^rd^ Trimester	Day 0 n = 327	Day 14 n = 82	Day 49 n = 112	Day 84 n = 115
Red Blood Cell (x10^6^/µl)	2.81–4.49	2.71–4.43	3.76 (0.52)	3.75 (0.72)	3.74 (0.53)	3.72 (0.40)
White Blood Cell (x10^9^/l)	5.6–14.8	5.9–16.9	7.59 (2.18)	8.16 (2.24)	7.63 (2.33)	8.00 (1.85)
Haemoglobin (g/dl)	9.7–14.8	9.5–15.0	10.86 (1.45)	10.93 (1.54)	10.90 (0.94)	11.07 (1.00)
Hematocrit (%)	30.0–39.0	28.0–40.0	29.68 (4.12)	30.48 (4.88)	30.57 (3.95)	30.44 (2.92)
Mean Corpuscular Volume (fl)	82–97	81–99	79.30 (6.62)	80.68 (10.65)	82.12 (6.85)	82.18 (6.09)
Mean Corpuscular Haemoglobin (pg/cell)	30–33	29–32	29.10 (2.89)	29.50 (3.02)	29.64 (2.83)	29.95 (2.35)
Mean Corpuscular Haemoglobin Concentration (g/dl)	32.3–37.9	33.9–37.5	36.65 (1.15)	39.97 (1.17)	36.06 (1.03)	36.43 (1.06)
Red Cell Distribution Width (%)	13.4–13.6	12.7–15.3	13.88 (1.64)	14.72 (2.43)	14.70 (3.00)	13.78 (2.61)
Platelet Count (x10^9^/l)	155–409	146–429	253.18 (73.99)	238.02 (57.72)	232.04 (60.01)	231.23 (62.64)
Iron Total (µmol/l)	7.9–31.9	5.4–34.6	14.58 (7.90)	23.74 (12.90)	22.79 (10.82)	32.41 (13.67)
Transferrin (g/l)	2.20–4.41	2.88–5.30	3.35 (0.67)	3.11 (0.69)	3.14 (0.62)	3.10 (0.66)
Transferrin Saturation (%)	10–44	5–37	21.83 (12.68)	37.90 (18.28)	35.08 (16.87)	49.14 (20.73)
Ferritin (ng/ml)	2–230	0–116	33.90 (35.24)	40.60 (23.86)	44.04 (32.16)	41.38 (23.58)
Alpha 1 Acid Glycoprotein (g/l)	Unknown	Unknown	0.65 (0.26)	0.55 (0.17)	0.42 (0.19)	0.44 (0.16)
C Reactive Protein (mg/l)	0.4–20.03	0.4–8.1	5.93 (13.28)	4.80 (4.48)	4.69 (5.28)	4.33 (3.48)
Soluble Transferrin Receptor (mg/l)	Unknown	Unknown	4.32 (2.48)	4.20 (4.43)	3.78 (1.30)	3.42 (1.05)
Soluble Transferrin Receptor: log Ferritin Index	Unknown	Unknown	3.86 (14.80)	2.85 (2.13)	2.55 (1.09)	2.40 (1.48)
Hepcidin (ng/ml)	Unknown	Unknown	4.57 (6.45)	8.46 (8.21)	10.22 (8.05)	9.93 (8.08)

Donors were enrolled (Day 0) between 14–22 weeks of gestation, making Days 0 and 14 fall in the 2^nd^ trimester, whereas Days 49 and 84 could be late 2^nd^ trimester or early 3^rd^ trimester depending on the individual. Tests were performed in MRC Keneba laboratories using a Medonic M20M GP and Cobas Integra 400 plus. Values in the normal range columns are the normal or healthy range for each parameter for 2^nd^ and 3^rd^ trimesters of pregnancy, as defined by standard guidelines^50^ if known. Numerical values reflect the mean value of all individuals and values in parentheses indicate standard deviation. Note that control non-anaemic, non-pregnant donors had an average haemoglobin of 14.13 g/dl (SD 0.85).

### *P. falciparum in vitro* growth rates are reduced in anaemic pregnant Gambian women

**Figure 2:**
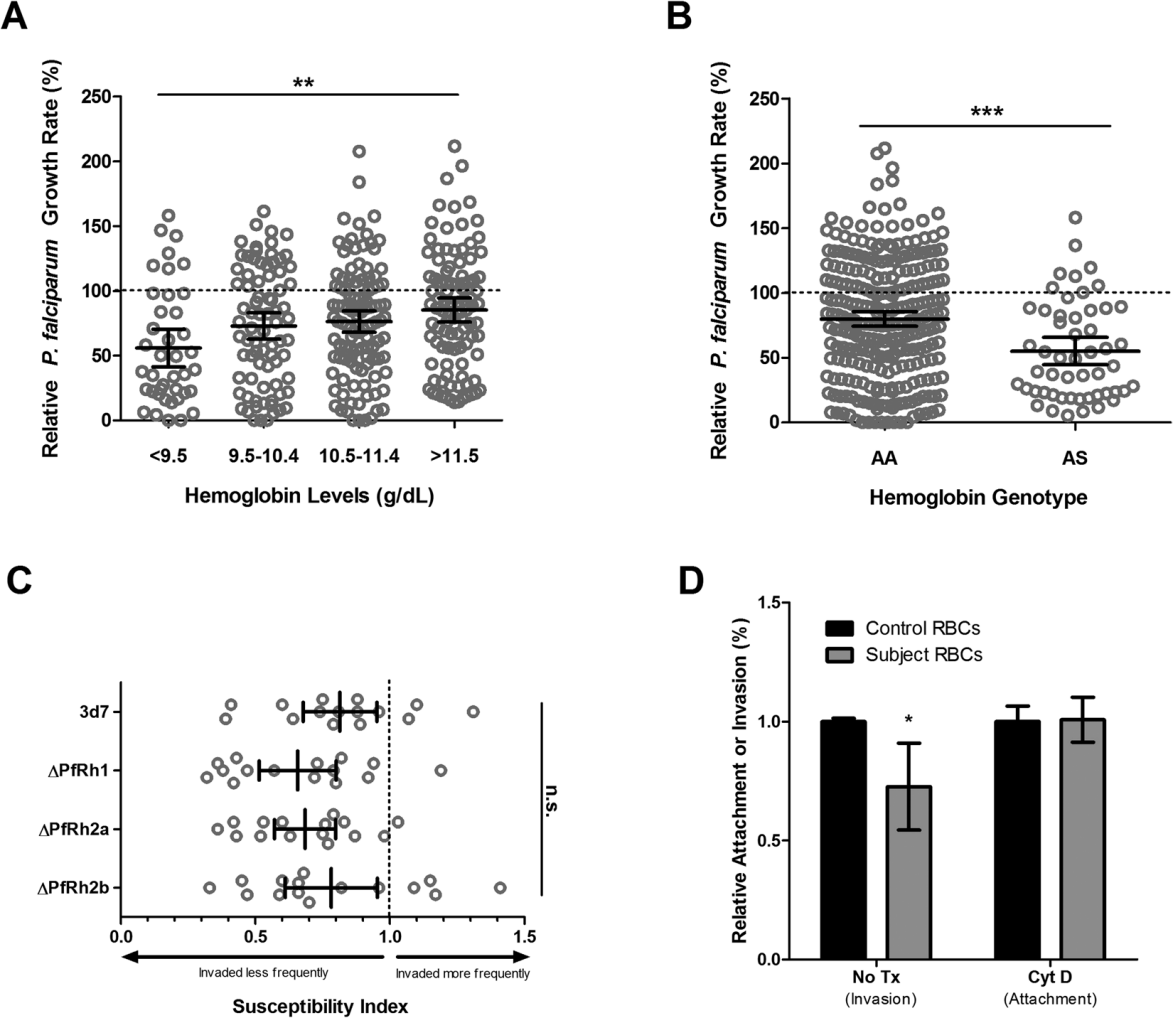
Parasite growth and invasion in RBCs from pregnant women (14–22 weeks gestation) prior to iron supplementation. (**A**) *P*. *falciparum* (strain FCR3-FMG) growth rates are proportional to haemoglobin concentration. Growth assays were performed in RBCs drawn from pregnant women at baseline (Day 0) and values are presented relative to growth in RBCs from non-anaemic, non-pregnant donors. Each dot represents the mean result of triplicate growth assays from each donor and the error bars represent 95% CI around the mean. One-way ANOVA indicates the means are different between Hgb levels (p < 0.01); specifically, post-hoc analysis with Tukey’s test indicates significant differences between Hgb levels <9.5 and >11.5 g/dl (**p < 0.01). (**B**) *P*. *falciparum* (strain FCR3-FMG) growth rates are reduced in RBCs from donors with sickle-cell trait compared to RBCs from donors with normal haemoglobin genotype. Growth assays were performed in RBCs drawn from pregnant women at baseline (Day 0) and values are presented relative to growth in RBCs from non-anaemic, non-pregnant donors with normal genotype. Each dot represents the mean result of triplicate growth assays from each donor and the error bars represent the 95% CI around the mean. AA indicates normal genotype and AS indicates sickle-cell trait genotype. Student’s t-test indicates the means are different between genotypes (***p < 0.001). (**C**) Competitive invasion of RBCs from anaemic (Hgb < 11 g/dl) and non-anaemic subjects using *P. falciparum* parent strain 3D7 and transgenic strains missing individual PfRh invasion ligands. Invasion experiments for RBCs from all test subject donors were performed independently and each experiment was performed in triplicate. Data show the mean SI using RBCs from the same 15 pregnant test donors for each strain. The SI defines the relative susceptibility to invasion of two different types of RBCs, control and test subject. The marker represents the SI point estimate and the bar represents the 95% CI. An SI of 1.0 indicates no difference in parasite invasion of two RBC populations. All strains give mean SI values significantly less than 1.0: 3D7 0.81 (95% CI 0.68–0.95), ΔPfRh1 0.66 (95% CI 0.51–0.80), ΔPfRh2a 0.68 (95% CI 0.57–0.80), ΔPfRh2b 0.78 (95% CI 0.61–0.95); however SI values were not significantly different from one another. (**D**) *P. falciparum* FCR3-FMG merozoite invasion into and attachment to RBCs from anaemic pregnant women (Hgb < 11 g/dl) compared to RBCs from non-anaemic, non-pregnant donors Purified schizonts were incubated with control or subject RBCs in parallel for 3–7 hrs to allow for schizont rupture. Subsequent fixation and staining with DNA dye allowed for detection of either new invasion events or attachment events, which are distinguishable from seeded schizont-infected RBCs by flow detection of DNA content. Attachment was specifically assayed with inclusion of Cytochalasin D during schizont incubation to prevent further invasion. New parasite events relative to starting parasitaemia were compared between subject and control RBCs (with rates in subject RBCs normalized to those in control RBCs within each experiment, in order to allow for inter-experiment comparisons). Results are shown from 2 experiments with RBCs from 3 control and 5 subject donors, each assayed in triplicate. Donors were chosen randomly, amongst those subjects from whom fresh RBCs were available on the day the necessary supplies and personnel were prepared to conduct the experiment. Error bars represent 95% CIs for the subject data. The mean invasion rate of subject RBCs relative to control RBCs was 0.62 (95% CI 0.49–0.76). The mean attachment rate for subject RBCs relative to control RBCs was 0.97 (95% CI 0.86–1.08). There is a significant difference in merozoite invasion in subject RBCs compared to control RBCs by Student’s t-test (*p < 0.05), but no difference in merozoite attachment.

First, we stratified by participant haemoglobin status at Day 0, and found malaria growth rates were progressively lower in the lower haemoglobin groups (mean growth rate (GR) compared to control GR of 100%: Hgb < 9.5 g/dl GR = 55.81%; Hgb 9.5–10.4 g/dl GR = 72.84%; Hgb 10.5–11.4 GR = 76.19%, Hgb > 11.5 g/dl GR = 85.23%; p < 0.01, one-way ANOVA) (Fig. 2A). Clear decreases in growth rates can also be seen between those with sickle-cell trait (AS) compared to those with normal haemoglobin (AA) genotype at Day 0 (when stratifying participants by haemoglobin genotype, mean GR compared to control GR of 100% was 79.84% for AA and 55.02% for AS, p < 0.001, Student’s t test) (Fig. 2B).

To examine the impact of haematological, iron, and inflammatory variables on parasite growth in RBCs from our study population, we performed bivariate and multivariate linear regression analyses. We assessed the influence of several key factors known or assumed to influence anaemia and/or parasite growth (Table 2). Haemoglobin, haemoglobin genotype, MCV, transferrin saturation (TSAT), as well as log-transformed hepcidin, RDW, and sTfR, all influenced parasite growth in RBCs drawn from pregnant women prior to iron supplementation. Transferrin, CRP, ferritin, MCH and gestational age did not. The only haemoglobin genotypes we were able to compare were normal (AA) and sickle-trait (AS). The growth rate increased 8.1% for every g/dl increase in haemoglobin and 0.9% increase for every 1 fl increase in MCV, for example, but decreased 24.8% in RBCs from donors with sickle-cell trait. The effect of haemoglobin on parasite growth was independent of haemoglobin genotype (Table 2), as well as other significant variables such as MCV, hepcidin, TSAT, RDW, ferritin, sTfR, CRP, and gestational age (data not shown).

**Table 2:** Effect of host haemoglobin, iron status, and other haematological characteristics on *in vitro P. falciparum* growth in RBCs from pregnant women (14–22 weeks gestation) at baseline.

Condition	B1 value	Lower CI	Upper CI	p value	Standardized % GR change
*Bivariate analysis of measures affecting parasite growth*					
Hgb (g/dl)	0.081	0.048	0.114	**<0.0001**	**11.70**
Hgb genotype (AA vs. AS)	−0.248	−0.383	−0.114	**<0.0001**	**−24.82**
MCV (fl)	0.009	0.002	0.017	**0.017**	**6.02**
MCH (pg/cell)	0.00	−0.01	0.02	0.585	
log Ferritin (ng/ml)	0.004	−0.119	0.128	0.944	
log Hepcidin (ng/ml)	0.086	0.022	0.150	**0.009**	**6.62**
log RDW (%)	1.805	0.787	2.824	**0.0006**	**9.03**
log CRP (mg/L)	0.033	−0.067	0.133	0.521	
log sTfR (mg/L)	−0.245	−0.487	−0.003	**0.048**	**−5.14**
Transferrin (g/L)	0.04	−0.04	0.11	0.367	
Transferrin saturation (%)	0.687	0.297	1.078	**0.0006**	**8.66**
Gestational age (weeks)	0.005	−0.015	0.025	0.626	
*Multivariate analysis of significant measures affecting parasite growth controlling for possible confounders*					
Hgb affects parasite growth controlling for Hgb genotype	0.076	0.042	0.109	**<0.0001**	**9.70**
Hgb genotype affects parasite growth controlling for Hgb	−0.227	−0.358	−0.096	**0.0008**	**−22.67**

Growth rates (GR) were calculated relative to growth in healthy, non-anaemic donors. Growth assays were performed in triplicate for each donor and the average value was used for linear regression modelling; multivariate analyses represent the estimated association for a given variable while controlling for potential confounders. Haemoglobin genotype was evaluated solely based on normal (AA) vs. sickle-cell trait (AS) classification. For continuous variables, the β1 value represents the %GR change for every 1 unit increase in the primary variable. For the categorical variable, the β1 value represents the %GR change based on yes-no genotype. For example, for Hgb AS, the %GR change is −24.8% relative to Hgb AA. Significant p values (p < 0.05) are bolded. The standardized %GR change for all continuous variables is calculated based on the SD for the exposure variable of interest (see Table 1) multiplied by β1 (x100%), to give the %GR change for every 1 SD change in the exposure variable; for Hgb genotype the standardized %GR change is simply β1 (x100%).

To better compare the effect size of haemoglobin, haemoglobin genotype, MCV, MCH, RDW, TSAT and log hepcidin, we calculated percentage GR change for every one standard deviation (SD) in the variable of interest. Hgb (as a continuous variable) has the largest effect on parasite growth, changing 11.7% with every increment of 1 SD. Sickle-cell trait (yes/no) also has a large impact on parasite growth (24.8% reduction) (Table 2).

Finally, we assessed haemoglobin as a categorical variable, as another way to compare the effect of haemoglobin on parasite growth. We assessed several different groupings of haemoglobin, trying to maintain clinical relevance (categorizing by anaemic and non-anaemic status (WHO criteria, CDC criteria, and adjusting for lower Hgb values in African populations); non-anaemic and mild and moderate anaemia (WHO criteria); and finally by every 1 g/dl change in haemoglobin). For all categorizations, haemoglobin maintained significant association with parasite growth rate at approximately the level seen using haemoglobin instead as a continuous variable (Supplemental Table 3).

### RBCs from anaemic pregnant women are resistant to invasion by *P. falciparum*, but invasion deficits are not attributable to PfRh invasion ligands or parasite attachment

We have previously shown that merozoites prefer iron replete RBCs from non-anaemic donors over RBCs from iron-deficient and anaemic donors^4,25,37^. Here we investigated the possible role of *P. falciparum* reticulocyte binding-like-homolog invasion ligands, PfRh1, PfRh2a or PfRh2b, in differential invasion of RBCs from anaemic donors (reviewed in^38^). Using a RBC barcoding invasion assay^37^, we directly examined merozoite invasion into differentially labelled target RBCs from anaemic pregnant women (15 donors) versus non-anaemic controls using reference laboratory strain 3D7 and three transgenic *P. falciparum* parasite strains, ΔPfRh1, ΔPfRh2a, and ΔPfRh2b, in parallel. The Susceptibility Index (SI) for the laboratory strain 3D7 was reduced (SI = 0.81), indicating reduced invasion into RBCs from anaemic donors. However, there were no differences in SI values between the parent (3D7) and transgenic parasite strains with altered PfRh expression. Mean SI values for each of the transgenic strains (ΔPfRh1, ΔPfRh2a, and ΔPfRh2b) were all below 1.0. This confirms merozoite invasion preference for RBCs from non-anaemic versus anaemic pregnant women, and demonstrates that PfRh1, PfRh2a and PfRh2b do not play in the RBC selection process (Fig. 2C).

We next examined whether merozoite attachment was altered during invasion of anaemic RBCs. To do so, we seeded parallel wells containing RBCs from control donors and anaemic pregnant women with late-stage *P*. *falciparum* strain FCR3-FMG schizonts in the presence and absence of Cytochalasin D, which prevents invasion steps beyond attachment. Thus we could differentiate invasion and attachment events by flow cytometry analysis^30^. Merozoites attached equally well to RBCs from anaemic and non-anaemic RBCs (Fig. 2D).

### *P. falciparum* growth rates increase in RBCs from pregnant women undergoing iron supplementation

**Figure 3:**
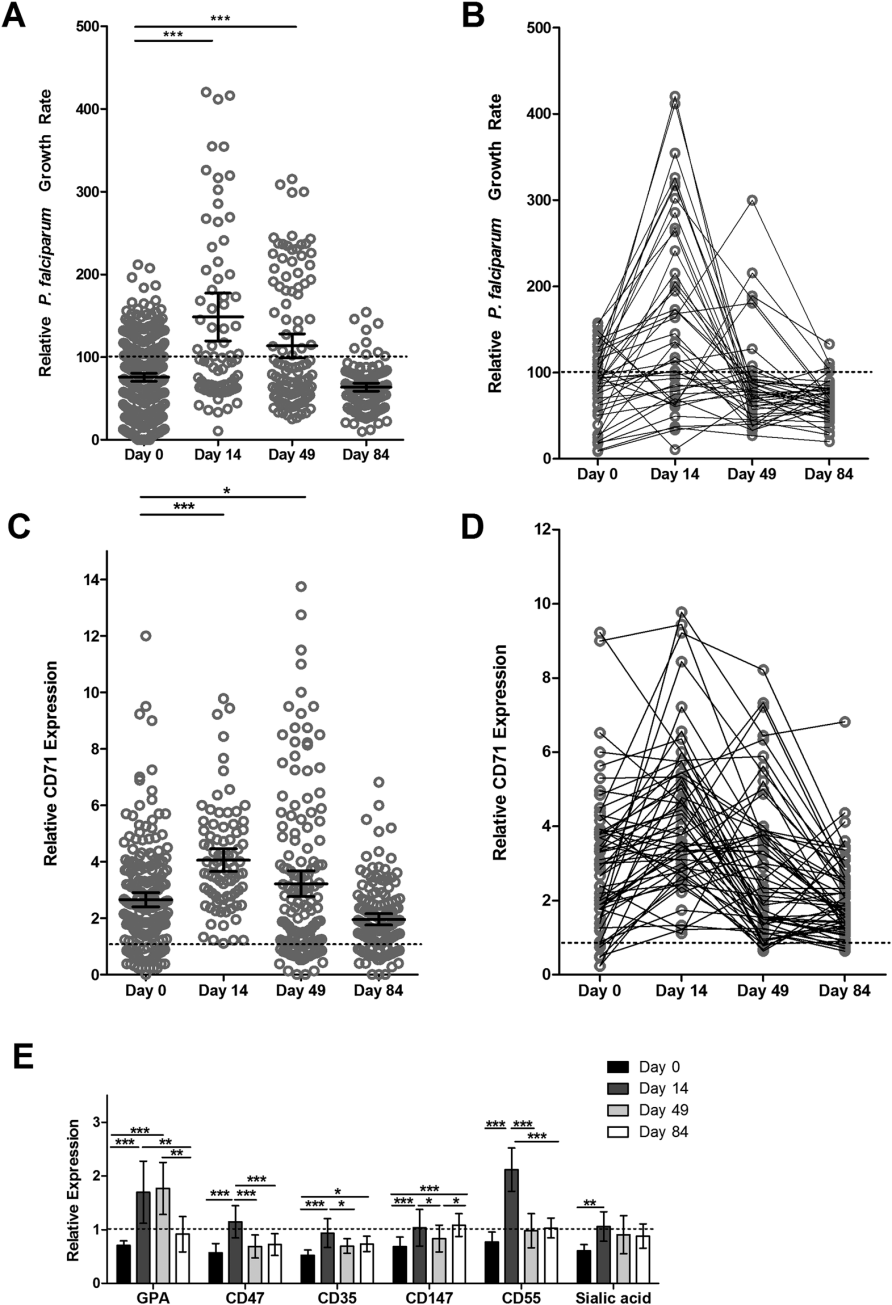
Malaria susceptibility increases transiently during iron supplementation and pregnant women receiving iron supplements have increased numbers of young RBCs. (**A**) *P. falciparum in vitro* growth rates in RBCs from pregnant women increase over time with iron supplementation (60 mg iron daily for 84 days). Parasite growth assays were conducted in RBCs from pregnant women at Day 0, Day 14, Day 49, and Day 84 using strain FCR3-FMG. Growth rates are reported relative to growth in RBCs from non-anaemic, non-pregnant donors (set at 100%). Each dot represents the mean of triplicate assays and error bars represent 95% CI around the mean. Differences between growth rates at the different time points were significant (p < 0.0001 by one-way ANOVA). Post-hoc analysis with Tukey’s test indicates significant differences between Days. Specifically, significant differences between Day 0 and Day 14 or Day 49 are highlighted in the graph (***p < 0.001). The only non-significant difference was between Day 0 and Day 84. n = 327 pregnant women at Day 0, n = 82 pregnant women at Day 14, n = 112 pregnant women at Day 49, and n = 115 pregnant women at Day 84. (**B**) Levels of parasite growth rates increase over time in pregnant women undergoing iron supplementation, as indicated by paired longitudinal data points. Line graph highlights changes for each individual that had data available at all time points (n = 44 pregnant women with complete repeat growth measures at Day 0, 14, 49, and 84 (average Day 0 haemoglobin = 10.84 g/dl), with 88.6% having increased growth rate at Day 14 and 54.5% having increased growth rate at Day 49. One-way repeated measures ANOVA of growth rate values indicates the means are significantly different between Days (p < 0.0001); post-hoc analysis with Tukey’s test indicates significant differences between Day 0 and Day 14 means (p < 0.001), but no significance between Day 0 and Day 49 or Day 84 for those pregnant women with repeat measures. (**C**) Levels of CD71-positive RBCs increase over time in pregnant women undergoing iron supplementation. Percent CD71-positive RBCs was measured by flow cytometry analysis of CD71 surface expression and is expressed relative to levels in RBCs from non-anaemic, non-pregnant controls (set at 1.0). Error bars represent the 95% CI; one-way ANOVA indicates the means are significantly different between Days (p < 0.0001). Post-hoc analysis with Tukey’s test indicates significant differences between Days. Significant differences between Day 0 and Day 14 or Day 49 are specifically highlighted in the graph (*p < 0.05, ***p < 0.001). n = 209 pregnant women at Day 0, n = 83 pregnant women at Day 14, n = 152 pregnant women at Day 49, and n = 142 pregnant women at Day 84. (**D**) Levels of circulating CD71-positive RBCs increase over time in pregnant women undergoing iron supplementation, as indicated by paired longitudinal data points. Line graph highlights changes for each individual that had data available at all time points (n = 60 pregnant women with complete repeat CD71 measures at Day 0, 14, 49, and 84). 78.3% at Day 14 and 46.7% at Day 49 had increased had increased CD71-positive RBCs versus Day 0. One-way repeated measures ANOVA of growth rate values indicates the means are significantly different between Days (p < 0.0001); post-hoc analysis with Tukey’s test indicates significant differences between Day 0 and Day 14 (p < 0.001). (**E**) Surface markers of RBC age and integrity change in a pattern consistent with an increase in erythropoiesis in pregnant women undergoing iron supplementation. Data represent relative expression based on subject donor RBC MFI values compared to RBCs from a non-anaemic, non-pregnant control donor not receiving iron supplementation (set at relative expression = 1.0). RBCs from the same 6 donors were examined over time. Error bars represent the 95% CI. One-way repeated measures ANOVA with post-hoc Tukey’s test analysis indicates significant difference between expression levels (*p < 0.05, **p < 0.01, ***p < 0.001).

In order to assess malaria susceptibility in individuals taking iron supplements, we performed *in vitro* growth assays in RBCs drawn from pregnant women at baseline (Day 0), during (Day 14 and Day 49) and at the end of (Day 84) 12 weeks of iron supplementation. Parasite growth rates were low at Day 0 (75.6%, 95% CI 70.6–80.6%), but increased markedly at Day 14 (148.5%, 95% CI 119.7–177.3%) and remained high at Day 49 (113.6%, 95% CI 99.16–128.1%), and finally returned to baseline values by Day 84 (63.62%, 95% CI 58.73–68.50%) (Fig. 3A). We also examined growth rate changes in RBCs from those individuals for whom we were able to obtain RBCs from at each time point (n = 44) and confirmed the same patterns of increased susceptibility over and above control levels of 100% at Day 14 and Day 49 (Fig. 3B).

### Pregnant women undergoing iron supplementation have increased levels of young RBCs in circulation

To evaluate the RBC population age structure of pregnant women undergoing daily iron supplementation, we measured levels of CD71-positive reticulocytes in RBCs drawn at baseline (Day 0), during (Day 14 and Day 49) and after (Day 84) iron supplementation (Fig. 3C). Paralleling changes in parasite growth rates, relative CD71 levels increased significantly from Day 0 (mean 2.65, 95% CI 2.40–2.91) to Day 14 (mean 4.06, 95% CI 3.65–4.46) and remained higher at Day 49 (mean 3.22, 95% CI 2.77–3.68), before returning to baseline levels at Day 84 (mean 1.96, 95% CI 1.76–2.15). One-way ANOVA indicates differences between the means are significant (p < 0.0001). If the analysis is limited to those pregnant women with values at all four time points (n = 60), the same pattern of increased young CD71-positive reticulocytes at Day 14 and Day 49 is observed (Fig. 3D).

We further examined other surface markers on RBCs from pregnant women undergoing iron supplementation over time (n = 5), in order to better understand changes in RBCs which may contribute to increased parasite growth rates following iron supplementation. We measured RBC surface proteins that are either known parasite invasion receptors and/or used to assess RBC age and membrane integrity. Specifically, we measured: (1) glycophorin A (GPA), an abundant RBC sialoglycoprotein which contributes to RBC surface charge, is found at higher levels on younger RBCs^39^, and is a merozoite receptor; (2) CD47, an anti-phagocytic marker which is at higher concentration on young RBCs^40^; (3) CD35 (complement receptor 1), which is more abundant on younger RBCs^41^; (4) CD147 (Basigin), the only known essential *P. falciparum* invasion receptor^42^; (5) CD55 (decay accelerating factor) which is involved in the complement system and has been shown to reflect RBC age^41^; and (6) sialic acid, which is reduced on older RBCs^40^. We again found significant increases from Day 0 to Day 14 (p < 0.001) for all surface markers examined, with the exception of sialic acid residues where the increase from Day 0 to Day 14 was slightly less significant (p < 0.01) (Fig. 3E). Some proteins measured also remained elevated at Day 49 and beyond, though the biggest changes were consistently measured between Day 0 and Day 14. Shifts in relative expression of these RBC markers are highly indicative of an overall increase in young, healthy RBCs.

## Discussion

Use of *in vitro* growth assays as our primary outcome provided an opportunity to systematically examine the cellular determinants of parasite growth in pregnant women. Here we show *P. falciparum* growth decreases in RBCs from anaemic (Hgb < 11 g/dl) pregnant Gambian women and that protection offered by anaemia in pregnant women in the second trimester of pregnancy is substantial (~11.7% change per standard deviation shift in haemoglobin). Of course, these results are from *in vitro* experiments that cannot fully capture disease protection dynamics in the real world, and we purposefully eliminated potentially confounding factors such as IPTp protection. However, our findings are consistent with many epidemiological studies observing reduced malaria infection rates in anaemic versus non-anaemic pregnant women^11–13^ and do provide additional insight into the degree of protection afforded by anaemia. Furthermore, our findings reveal parasite growth in RBCs from pregnant Gambian women directly correlates with other markers of iron deficiency such as MCV, TSAT, hepcidin, and sTfR. Overall, this parasite growth and linear regression data is highly consistent with the data we have previously reported on anaemic Gambian children^29^.

The molecular mechanisms by which RBCs from anaemic pregnant women are protected remain unknown. We examined whether specific *P. falciparum* PfRh invasion ligands contribute to the differential invasion. *P. falciparum*’s multiple invasion ligands represent different invasion pathways which the parasite can utilize to enter RBCs (reviewed^38^). We hypothesized that PfRh ligands may help the parasite detect the healthiest RBCs through their putative ATP binding domains^43,44^. However, we found no differences between the wild type and the transgenic parasite strains missing either PfRh1, PfRh2a or PfRh2b. We next examined whether merozoite attachment was affected during invasion of anaemic RBCs and again found no differences. Both of these sets of experiments are consistent with the hypothesis that the mechanical properties of RBC membranes from anaemic donors^45,46^ influence invasion. Recent work on merozoite invasion has demonstrated that localized RBC deformation is important for parasite invasion^47,48^.

Finally, we demonstrate that parasite growth increased dramatically relative to baseline in RBCs taken from pregnant women during iron supplementation, transiently rising as early as 14 days after iron supplementation was initiated. The growth remained high at Day 49 relative to non-anaemic controls and returned by Day 84 to baseline levels. These patterns in growth rate changes were paralleled by measurements of markers of young RBCs in circulation, specifically CD71-positive reticulocytes together with other markers of RBC age such as GPA and CD47. While the majority of individuals’ growth rates peaked at Day 14, some peaked at Day 49. Differences in gestational age and other physiological factors that may influence RBC properties and differences in RBC population dynamics in response to iron administration may cause this. The rapid increase in both parasite growth and young RBC populations reflect a shift that can be observed quite quickly in pregnant women already subject to physiological erythrocytic drive. Pregnant women naturally have elevated levels of erythropoietin, which drives a blood volume and RBC mass expansion. Similarly, it is not surprising that CD71 levels in pregnant women are generally higher than in non-anaemic, non-pregnant control donors at baseline, as pregnant women have an expanding erythroid mass with increased levels of erythropoietin. These results are consistent with a report from Tian *et al*. who show that parasites grow better in RBCs from pregnant women (n = 9) in their 2^nd^ trimester compared to non-pregnant women (n = 9)^49^.

One of the important limitations of this study is that it does not include a group of women who did not receive iron supplements and therefore there is no way to separate the impact of pregnancy alone on malaria growth from the impact of iron supplementation. Nonetheless, whether or not significant haemoglobin increases are induced by iron supplementation or natural haematological changes occurring between the 2^nd^ and 3^rd^ trimesters of pregnancy, the changing erythropoietic drive seems clearly linked to parasite growth increases *in vitro* and thus reflects a real concern for increased malaria susceptibility in pregnant women, particularly those taking iron. Another significant limitation is that we are using *in vitro* parasite growth data as a surrogate marker for malaria susceptibility. However, this approach has allowed us to test RBCs from over 400 anaemic and non-anaemic pregnant women on whom we had complete iron and inflammatory marker data. This has also allowed us examine the impact of RBCs on malaria susceptibility separate from the effects of IPTp and the immune response to infection.

Implementation rates of IPTp-SP are currently low in malarious areas; only about 25% of pregnant women in Sub-Saharan Africa receive two doses of IPTp^24^. With low IPTp-SP uptake, our *in vitro* assays conducted in the absence of confounders help to increase understanding of the host-pathogen dynamics. In addition, our demonstration that there are profound increases in parasite invasion and growth rates in RBCs within 2 weeks of starting iron therapy provide compelling evidence to strengthen WHO’s existing recommendation that, where *P. falciparum* is endemic, iron supplementation should be given in combination with measures to prevent, monitor and treat malaria in pregnancy (primarily through provision of monthly IPTp-SP and access to adequate health care).

## Electronic supplementary material

Supporting Information

## References

[1] Stevens GA (2013). Global, regional, and national trends in haemoglobin concentration and prevalence of total and severe anaemia in children and pregnant and non-pregnant women for 1995–2011: a systematic analysis of population-representative data. Lancet Glob. Health.

[2] Di Renzo GC (2015). Iron deficiency anaemia in pregnancy. Womens Health Lond. Engl..

[3] Ansell J, Hamilton KA, Pinder M, Walraven GEL, Lindsay SW (2002). Short-range attractiveness of pregnant women to Anopheles gambiae mosquitoes. Trans. R. Soc. Trop. Med. Hyg..

[4] Clark MA (2014). Host iron status and iron supplementation mediate susceptibility to erythrocytic stage Plasmodium falciparum. Nat. Commun..

[5] Lim C (2013). Expansion of host cellular niche can drive adaptation of a zoonotic malaria parasite to humans. Nat. Commun..

[6] Duffy PE, Fried M (2003). Plasmodium falciparum adhesion in the placenta. Curr. Opin. Microbiol..

[7] Fried M, Duffy PE (1996). Adherence of Plasmodium falciparum to chondroitin sulfate A in the human placenta. Science.

[8] Ricke CH (2000). Plasma antibodies from malaria-exposed pregnant women recognize variant surface antigens on Plasmodium falciparum-infected erythrocytes in a parity-dependent manner and block parasite adhesion to chondroitin sulfate A. J. Immunol. Baltim. Md 1950.

[9] Desai M (2007). Epidemiology and burden of malaria in pregnancy. Lancet Infect. Dis..

[10] WHO | WHO recommendations on antenatal care for a positive pregnancy experience. WHO Available at: http://www.who.int/reproductivehealth/publications/maternal_perinatal_health/anc-positive-pregnancy-experience/en/ (Accessed: 10th February 2017).

[11] Kabyemela ER, Fried M, Kurtis JD, Mutabingwa TK, Duffy PE (2008). Decreased susceptibility to Plasmodium falciparum infection in pregnant women with iron deficiency. J. Infect. Dis..

[12] Senga EL, Harper G, Koshy G, Kazembe PN, Brabin BJ (2011). Reduced risk for placental malaria in iron deficient women. Malar. J..

[13] Adam I, Ehassan EM, Mohmmed AA, Salih MM, Elbashir MI (2012). Decreased susceptibility to placental malaria in anaemic women in an area with unstable malaria transmission in central Sudan. Pathog. Glob. Health.

[14] Jonker FAM (2012). Iron status predicts malaria risk in Malawian preschool children. PloS One.

[15] Gwamaka M (2012). Iron deficiency protects against severe Plasmodium falciparum malaria and death in young children. Clin. Infect. Dis. Off. Publ. Infect. Dis. Soc. Am..

[16] Nyakeriga AM (2004). Iron deficiency and malaria among children living on the coast of Kenya. J. Infect. Dis..

[17] Murray MJ, Murray AB, Murray MB, Murray CJ (1978). The adverse effect of iron repletion on the course of certain infections. Br. Med. J..

[18] Murray MJ, Murray NJ, Murray AB, Murray MB (1975). Refeeding-malaria and hyperferraemia. Lancet.

[19] Oppenheimer SJ (1986). Iron supplementation increases prevalence and effects of malaria: report on clinical studies in Papua New Guinea. Trans. R. Soc. Trop. Med. Hyg..

[20] Smith AW, Hendrickse RG, Harrison C, Hayes RJ, Greenwood BM (1989). Iron-deficiency anaemia and its response to oral iron: report of a study in rural Gambian children treated at home by their mothers. Ann. Trop. Paediatr..

[21] Veenemans J (2011). Effect of supplementation with zinc and other micronutrients on malaria in Tanzanian children: a randomised trial. PLoS Med..

[22] Sazawal S (2006). Effects of routine prophylactic supplementation with iron and folic acid on admission to hospital and mortality in preschool children in a high malaria transmission setting: community-based, randomised, placebo-controlled trial. Lancet Lond. Engl..

[23] WHO | WHO policy brief for the implementation of intermittent preventive treatment of malaria in pregnancy using sulfadoxine-pyrimethamine (IPTp-SP). WHO Available at: http://www.who.int/malaria/publications/atoz/policy_brief_iptp_sp_policy_recommendation/en/ (Accessed: 10th February 2017).

[24] Hill J (2013). Factors affecting the delivery, access, and use of interventions to prevent malaria in pregnancy in sub-Saharan Africa: a systematic review and meta-analysis. PLoS Med..

[25] Goheen MM (2016). Anaemia Offers Stronger Protection Than Sickle Cell Trait Against the Erythrocytic Stage of Falciparum Malaria and This Protection Is Reversed by Iron Supplementation. EbioMedicine.

[26] Bah A (2016). A double blind randomised controlled trial comparing standard dose of iron supplementation for pregnant women with two screen-and-treat approaches using hepcidin as a biomarker for ready and safe to receive iron. BMC Pregnancy Childbirth.

[27] Triglia T, Duraisingh MT, Good RT, Cowman AF (2005). Reticulocyte-binding protein homologue 1 is required for sialic acid-dependent invasion into human erythrocytes by Plasmodium falciparum. Mol. Microbiol..

[28] Duraisingh MT (2003). Phenotypic variation of Plasmodium falciparum merozoite proteins directs receptor targeting for invasion of human erythrocytes. EMBO J..

[29] Goheen MM (2016). Anaemia Offers Stronger Protection Than Sickle Cell Trait Against the Erythrocytic Stage of Falciparum Malaria and This Protection Is Reversed by Iron Supplementation. EBioMedicine.

[30] Paul AS (2015). Parasite Calcineurin Regulates Host Cell Recognition and Attachment by Apicomplexans. Cell Host Microbe.

[31] Rothman, K. J., Greenland, S. & Lash, T. L. *Modern Epidemiology*. (Lippincott Williams & Wilkins, 2008).

[32] Goonewardene M, Shehata M, Hamad A (2012). Anaemia in pregnancy. Best Pract. Res. Clin. Obstet. Gynaecol..

[33] WHO | Haemoglobin concentrations for the diagnosis of anaemia and assessment of severity. *WHO* Available at: http://www.who.int/vmnis/indicators/haemoglobin/en/ (Accessed: 16th October 2016).

[34] Centers for Disease Control. CDC criteria for anaemia in children and childbearing-aged women. *MMWR Morb. Mortal. Wkly. Rep*. **38**, 400–4 (1989). 2542755

[35] Institute of Medicine. Iron Deficiency Anaemia: Recommended Guidelines for the Prevention, Detection, and Management Among U.S. *Children and Women of Childbearing**Age*. (The National Academies Press, 1993). 25144105

[36] Bah A (2017). Serum Hepcidin Concentrations Decline during Pregnancy and May Identify Iron Deficiency: Analysis of a Longitudinal Pregnancy Cohort in The Gambia. J. Nutr..

[37] Clark MA (2014). RBC barcoding allows for the study of erythrocyte population dynamics and P. falciparum merozoite invasion. PloS One.

[38] Bei AK, Duraisingh MT (2012). Functional analysis of erythrocyte determinants of Plasmodium infection. Int. J. Parasitol..

[39] Beeson JG (2016). Merozoite surface proteins in red blood cell invasion, immunity and vaccines against malaria. FEMS Microbiol. Rev..

[40] Lutz HU (2004). Innate immune and non-immune mediators of erythrocyte clearance. Cell. Mol. Biol. Noisy–Gd. Fr..

[41] Gwamaka M, Fried M, Domingo G, Duffy PE (2011). Early and extensive CD55 loss from red blood cells supports a causal role in malarial anaemia. Malar. J..

[42] Crosnier C (2011). Basigin is a receptor essential for erythrocyte invasion by Plasmodium falciparum. Nature.

[43] Ramalingam JK, Hunke C, Gao X, Grüber G, Preiser PR (2008). ATP/ADP binding to a novel nucleotide binding domain of the reticulocyte-binding protein Py235 of Plasmodium yoelii. J. Biol. Chem..

[44] Gunalan K, Gao X, Yap SSL, Huang X, Preiser PR (2013). The role of the reticulocyte-binding-like protein homologues of Plasmodium in erythrocyte sensing and invasion. Cell. Microbiol..

[45] Yip R (1983). Red cell membrane stiffness in iron deficiency. Blood.

[46] Brandão MM (2009). Impaired red cell deformability in iron deficient subjects. Clin. Hemorheol. Microcirc..

[47] Weiss GE (2015). Revealing the sequence and resulting cellular morphology of receptor-ligand interactions during Plasmodium falciparum invasion of erythrocytes. PLoS Pathog..

[48] Sisquella, X. *et al*. P. falciparum ligand binding to erythrocytes induce alterations in deformability essential for invasion. *eLife* **6** (2017). 10.7554/eLife.21083 PMC5333951 28226242

[49] Tian LP (1998). Red cell age and susceptibility to malaria during pregnancy. Acta Obstet. Gynecol. Scand..

[50] Bauer, K. A. Hematologic changes in pregnancy. *UpToDate* Topic 429 Version 16.0 (2016).

