# Supplementary material for: Host iron status and erythropoietic response to iron supplementation determines susceptibility to the RBC stage of falciparum malaria during pregnancy
Source: Sci Rep. 2017 Dec 15;7:17674. doi: 10.1038/s41598-017-16896-z (PMC5732269; doi:10.1038/s41598-017-16896-z)
Supplement: Supplementary file 1 — Supporting Information [file 41598_2017_16896_MOESM1_ESM.pdf]

Host iron status and erythropoietic response to iron supplementation determines susceptibility to the RBC stage of falciparum malaria during pregnancy

Running title: Iron and malaria in pregnancy

Morgan M. Goheen[a], Amat Bah[b], Rita Wegmuller[b], Hans Verhoef[cd], Bakary Darboe[b], Ebrima Danso[b], Andrew M. Prentice[b,c], Carla Cerami[b*]

[a] Department of Microbiology and Immunology, University of North Carolina School of Medicine, Chapel Hill, NC, USA

[b] Nutrition Theme, MRC Unit The Gambia, MRC International Nutrition Group, Keneba, The Gambia

[c] London School of Hygiene & Tropical Medicine, London, UK

[d] Division of Human Nutrition and Cell Biology and Immunology Group, Wageningen University, Wageningen, The Netherlands

*Corresponding author

Email: ccerami@mrc.gm

Supplemental Table Legends

Supplemental Table 1: Description of subjects at baseline.

Hemoglobin genotyping was performed by electrophoresis. *A portion of subjects (% of total) was unable to be tested for hemoglobin genotype so these subjects are not included in the denominator for other genotypes (which denote % of those tested).

Supplemental Table 2: Blood, inflammatory, and iron parameters of study participants with reportable parasite growth rate data versus those without.

Numerical values reflect the mean value of all individuals of a particular category and time point, and values in parentheses are the SD.

Supplemental Table 3: **Linear regression analysis using hemoglobin categorizations.**

To more thoroughly assess the impact of hemoglobin levels on parasite growth rate, hemoglobin was binned based on standard definitions of anemia in the second trimester of pregnancy. All $\beta 1$ values were significant ($p<0.01$ or greater). Significant p values ($p<0.05$) are bolded. The %GR change is simply $\beta 1$ (x100%).

Supplemental Tables

Supplemental Table 1

**Characteristic**		**All participants (n=498)**	**Those to receive 60 mg iron daily (n=167)**
Age, years, mean (SD)		27.1 (5.8)	27.0 (5.9)
Gravida, mean (SD)		3.6 (2.3)	3.6 (2.3)
Gestational age at enrollment, weeks, mean (SD)		19.9 (6.1)	19.4 (6.8)
Hemoglobin genotype, n (%)			
	AA	400 (82.3)	129 (79.6)
	AS	86 (17.7)	33 (20.4)
	SS	0	0
	AC	0	0
	SC	0	0
	Untested*	12 (2.4)	5 (3.0)

Supplemental Table 2

Variable	Day 0 with GR (n=327)	Day 0 no GR (n=172)	Day 14 with GR (n=82)	Day 14 no GR (n=83)	Day 49 with GR (n=112)	Day 49 no GR (n=45)	Day 84 with GR (n=115)	Day 84 no GR (n=29)
Red Blood Cell (x10^6 per µl)	3.76 (0.52)	3.73 (0.48)	3.75 (0.72)	3.55 (0.36)	3.74 (0.53)	3.56 (0.36)	3.72 (0.40)	3.55 (0.43)
White Blood Cell (x10^9 per l)	7.59 (2.18)	7.14 (1.89)	8.16 (2.24)	7.39 (1.85)	7.63 92.33)	7.22 (1.89)	8.00 (1.85)	8.63 (2.47)
Hemoglobin (g per dl)	10.86 (1.45)	10.61 (1.32)	10.93 (1.54)	10.44 (0.96)	10.90 (0.94)	10.72 (1.06)	11.07 (1.00)	10.74 (1.22)
Hematocrit (%)	29.68 (4.12)	29.65 (3.62)	30.48 (4.88)	28.49 (2.61)	30.57 (3.95)	29.23 (3.05)	30.44 (2.92)	29.74 (3.52)
Mean Corpuscular Volume (fl)	79.30 (6.62)	78.89 (7.44)	80.68 (10.65)	80.56 (6.51)	82.12 (6.85)	82.26 (5.12)	82.18 (6.09)	84.19 (7.93)
Mean Corpuscular Hemoglobin (pg per cell)	29.10 (2.89)	28.71 (3.13)	29.50 (3.02)	29.62 (2.83)	29.64 (2.83)	30.25 (2.12)	29.95 (2.35)	30.48 (3.03)
Mean Corpuscular Hgb Concentration (g per dl)	36.65 (1.45)	36.33 (0.96)	39.97 (1.17)	36.72 (1.11)	36.06 (1.03)	36.77 (1.06)	36.43 (1.06)	36.2 (0.80)
Red Cell Distribution Width (%)	13.88 (1.64)	13.51 (1.68)	14.72 (2.43)	14.00 (1.89)	14.70 (3.00)	13.70 (1.83)	13.78 (2.61)	13.45 (1.04)
Platelet Count (x10^9 per l)	253.18 (73.99)	262.72 (67.88)	238.02 (57.72)	268.86 (72.54)	232.04 (66.01)	232.76 (71.75)	231.23 (62.64)	231.46 (65.21)
Iron Total (µ mol per l)	14.58 (7.90)	15.73 (6.97)	23.74 (12.90)	22.14 (12.68)	22.79 (10.82)	16.92 (7.78)	32.41 (13.67)	34.01 (12.06)
Transferrin (g per l)	3.35 (0.67)	3.23 (0.60)	3.11 (0.69)	2.92 (0.70)	3.14 (0.62)	2.47 (0.43)	3.10 (0.66)	3.32 (0.50)
Transferrin Saturation (%)	18.6 (12.6)	23.8 (12.7)	38.0 (18.3)	34.7 (18.1)	35.1 (16.9)	27.7 (12.6)	49.1 (20.7)	48.6 (18.1)
Ferritin (ng per ml)	33.90 (35.24)	36.35 (50.01)	40.60 (23.86)	50.02 (42.18)	44.04 (32.16)	41.24 (38.33)	41.38 (23.58)	38.88 (20.17)
Alpha 1 Anti-glycoprotein (g per l)	0.65 (0.26)	0.68 (0.27)	0.55 (0.17)	0.57 (0.18)	0.42 (0.19)	0.39 (0.17)	0.44 (0.16)	0.50 (0.37)
C Reactive Protein (mg per L)	5.93 (13.28)	5.95 (11.89)	4.80 (4.48)	5.96 (7.78)	4.69 (5.28)	4.62 (4.99)	4.33 (3.48)	5.12 (5.41)
Soluble Transferrin Receptor (mg per l)	4.32 (2.48)	4.58 (2.06)	4.20 (4.43)	3.99 (1.63)	3.78 (1.30)	3.18 (1.06)	3.42 (1.05)	3.38 (0.96)
Soluble Transferrin Receptor: log Ferritin Index	3.85 (14.80)	3.56 (8.91)	2.85 (2.13)	2.62 (1.37)	2.55 (1.09)	2.28 (1.08)	2.40 (1.48)	2.26 (0.92)
Hepcidin (ng per ml)	4.57 (6.45)	7.75 (8.87)	8.46 (8.21)	8.65 (8.33)	10.22 (8.05)	8.71 (7.98)	9.93 (8.08)	9.43 (6.70)

Supplemental Table 3

**Condition**	**β value**	**Lower CI**	**Upper CI**	**p value**	**%GR change**
Hgb continuous (g/dl)	0.081	0.048	0.114	**<0.0001**	8.07
Hgb dichotomized (<11 or ≥11 g/dl)	0.151	0.054	0.249	**0.003**	15.14
Hgb dichotomized (<10.5 or ≥10.5 g/dl)	0.133	0.032	0.235	**0.011**	13.35
Hgb dichotomized (<9.7 or ≥9.7 g/dl)	0.242	0.106	0.378	**0.001**	24.21
Hgb tertiles (<10, 10-11, and ≥11 g/dl)	0.097	0.037	0.158	**0.002**	9.75
Hgb quartiles (<10, 10-11, 11-12, and ≥12 g/dl)	0.085	0.038	0.133	**0.001**	8.55
Hgb quintiles (<9, 9-10, 10-11, 11-12, and ≥12 g/dl)	0.079	0.037	0.121	**0.0003**	7.91
